# Preparing a neuropediatric upper limb exergame rehabilitation system for home-use: a feasibility study

**DOI:** 10.1186/s12984-016-0141-x

**Published:** 2016-03-23

**Authors:** Corinna N. Gerber, Bettina Kunz, Hubertus J. A. van Hedel

**Affiliations:** Pediatric Rehab Research Group, Rehabilitation Center for Children and Adolescents, Mühlebergstrasse 104, CH-8910 Affoltern am Albis, Switzerland; Department of Health Sciences and Technology, ETH Zurich, Rämistrasse 101, CH-8092 Zurich, Switzerland; Children’s Research Center, University Children’s Hospital Zurich, Steinwiesstrasse 75, CH-8032 Zürich, Switzerland

**Keywords:** Data glove, Pediatrics, Neurorehabilitation, Upper extremities, YouGrabber, Tele-rehabilitation, Game-based, Cerebral palsy, Children and adolescents, Clinical utility, User satisfaction

## Abstract

**Background:**

Home-based, computer-enhanced therapy of hand and arm function can complement conventional interventions and increase the amount and intensity of training, without interfering too much with family routines. The objective of the present study was to investigate the feasibility and usability of the new portable version of the YouGrabber® system (YouRehab AG, Zurich, Switzerland) in the home setting.

**Methods:**

Fifteen families of children (7 girls, mean age: 11.3y) with neuromotor disorders and affected upper limbs participated. They received instructions and took the system home to train for 2 weeks. After returning it, they answered questions about usability, motivation, and their general opinion of the system (Visual Analogue Scale; 0 indicating worst score, 100 indicating best score; ≤30 not satisfied, 31–69 average, ≥70 satisfied). Furthermore, total pure playtime and number of training sessions were quantified. To prove the usability of the system, number and sort of support requests were logged.

**Results:**

The usability of the system was considered average to satisfying (mean 60.1–93.1). The lowest score was given for the occurrence of technical errors. Parents had to motivate their children to start (mean 66.5) and continue (mean 68.5) with the training. But in general, parents estimated the therapeutic benefit as high (mean 73.1) and the whole system as very good (mean 87.4). Children played on average 7 times during the 2 weeks; total pure playtime was 185 ± 45 min. Especially at the beginning of the trial, systems were very error-prone. Fortunately, we, or the company, solved most problems before the patients took the systems home. Nevertheless, 10 of 15 families contacted us at least once because of technical problems.

**Conclusions:**

Despite that the YouGrabber® is a promising and highly accepted training tool for home-use, currently, it is still error-prone, and the requested support exceeds the support that can be provided by clinical therapists. A technically more robust system, combined with additional attractive games, likely results in higher patient motivation and better compliance. This would reduce the need for parents to motivate their children extrinsically and allow for clinical trials to investigate the effectiveness of the system.

**Trial registration:**

ClinicalTrials.gov NCT02368223

**Electronic supplementary material:**

The online version of this article (doi:10.1186/s12984-016-0141-x) contains supplementary material, which is available to authorized users.

## Background

Cerebral Palsy (CP) is the most common cause for motor disabilities in children in Western Countries (prevalence in Europe: 1.77/1’000 per year) [1]. Traumatic brain injuries (TBI) in children and youths (worldwide incidence of hospitalizations: 74/100’000 per year) [2], and childhood stroke (worldwide incidence: 1.2-13/100’000 children per year) [3] are other common causes for hospitalization with consecutive rehabilitation.

Many of these patients exhibit upper limb impairments such as reduced movement speed, finger dexterity, muscle strength, or interlimb coordination. As a consequence, these children might experience severe reductions in their independence in activities of daily living (ADL). In fact, one of the most important goals for these patients is to become more independent in their daily life [4]. Therefore, neuropediatric rehabilitation targets at reducing functional limitation and improving motor capacity and performance to achieve the best possible level of independence in daily life.

For successful neurorehabilitation, it is important, amongst others, that patients participate actively in therapeutic sessions, are challenged, motivated and rewarded, and that treatment is tailored to the patient’s needs [5]. Furthermore, a higher therapy dosage seems to lead to better motor outcomes [5, 6].

To optimize pediatric neurorehabilitation and to complement conventional occupational therapies, computer-based therapies for upper limb rehabilitation have been developed or adapted for the pediatric field during recent years [4]. The natural play instinct evoked by the computer games is thought to lead to higher motivation and engagement [7] and, therefore, could lead to more effective rehabilitation. In their meta-analysis, Chen et al. (2014) found a strong effect size for computer-enhanced interventions in children with CP [8].

Such patient-tailored rehabilitation programs (including or without computer-enhanced therapy systems) require considerable time commitments of therapists and are therefore expensive [4]. After discharge from an inpatient rehabilitation stay, where these patients receive an intensive interdisciplinary rehabilitation program, the consecutive out-patient program is often much less intensive, with 1–2 h of therapy sessions per week at the most. This frequency is not considered sufficient to achieve optimal, long-lasting improvements [5]. Therefore, the interest for effective, sophisticated, preferably low-cost, home-based training systems allowing an intensive, motivating training over a longer time, is constantly growing.

The gold standard until now remains the 1:1 session with a skilled therapist and the content tailored to the everyday needs of the patients. Home-based, therapeutic exergaming could complement this therapy. The therapy is applied directly in the patient’s home environment, which could make it available for more children who might benefit from such an intervention because it can be easier embedded in the family’s daily routines [8] and it saves travel time of children and their caregivers [8, 9]. The therapy planning is more flexible, and the amount and intensity of training can be better controlled [9] and augmented [10]. It further allows continuity of patient care [8, 9] and, ideally, such therapy-systems should not need constant surveillance by a therapist, which might even reduce treatment costs [11]. Overall, home-based systems might reduce the burden on the family.

Additionally to the requirement of being effective and safe, therapy systems for home-use should be handy and require little space as they have to be transported to and installed in the patient’s home. Furthermore, they should be easy to set-up and user-friendly as patients and their families should use them without the support of a therapist or technician. However, to supervise the device usage from time to time and adjust training programs e.g. for adjustment of exercise difficulties, the therapist should have the possibility to remotely control the device. Moreover, users should have access to technical support when problems occur.

There are also important clinical requirements that computer-based systems for home-use should fulfill. They have to allow tailor-made therapy adjusted to the patient’s abilities and needs. Motor learning principles from basic science such as active patient participation, challenging exercises, high movement repetition with enough variations, patient motivation, and reward should be met [5]. Of course, clinical trials should provide evidence that the systems are effective.

The goal of our study was to test home-based computer-enhanced upper limb training with the YouGrabber (YouRehab AG, Zurich, Switzerland) for children and adolescents with central motor disorders. For this purpose, the system was given to the patient’s home for two consecutive weeks. Specific aims were to determine (i) the feasibility of the YouGrabber system in a home setting with minimal supervision, (ii) the acceptance of the system as well as the motivation for using it, and (iii) the usability of the device.

## Methods

### Setting and participants

The ethics committee of the Canton of Zurich, Switzerland approved the study (KEK-StV 24/07). Written informed consent was obtained from the legal guardians of all participating children before inclusion in the study. Adolescents aged 15 years and above provided written informed consent while younger children provided oral agreement.

A convenience sample of in- and outpatients of the rehabilitation center for children and adolescents in Affoltern am Albis, Switzerland was recruited between February and July 2015. Children and adolescents were included if they met the following criteria: (i) aged 5–18 years, (ii) central motor disorders involving at least one upper extremity, (iii) Manual Ability Classification System [12] (MACS)-level I-IV, (iv) capability to understand and follow the instructions, (v) ability to sit in an upright position for a minimum of 45 min, (vi) full weight bearing of the upper extremities, and (vii) internet access at home. Exclusion criteria were: (i) severe visual or auditory problems (ii) children or parents with insufficient knowledge of the German language, (iii) MACS-level V, (iv) open skin lesions on the hands/arms, (v) severe photosensitive epilepsy. Patient Characteristics are shown in Table 1.

**Table 1:** Patient characteristics

ID (phase)	Age [y]	Gender	Diagnosis	Affected side	MACS-level	WeeFIM sc [%]	TONI-4 [%]
01 (I)	12.2	m	CP	left arm	III	81.0	12
02 (I)	14.8	f	Neuropathy	bilateral	II	97.6	45
03 (I)	13.6	m	CP	bilateral	II	76.2	3
04 (I)	14.5	m	GA1	bilateral	III	66.7	61
05 (I)	6.1	m	CP	bilateral	II	65.8	61
06 (II)	18.5	f	CP	bilateral	II	92.9	10
07 (II)	9.4	m	CP	bilateral	I	61.9	63
08 (II)	9.8	f	CP	bilateral	I	95.2	55
09 (II)	8.8	f	CP	bilateral	III	54.8	6
10 (II)	7.8	f	CP	bilateral	II	71.8	16
11 (I)	13.0	m	CP	bilateral	I	100.0	84
12 (II)	14.3	m	CP	bilateral	I	78.6	73
13 ^a^ (II)	14.8	f	CP	bilateral	II	81.6	NA
14 (II)	6.0	m	CP	right arm	II	86.8	NA
15 (II)	6.1	f	CP	left arm	II	97.4	NA
Mean (SD)	11.3 (3.9)					80.6 (14.3)	40.8 (29.3)

Abbreviations: *ID* Identification, *MACS* Manual Ability Classification System, *WeeFIM sc* Functional Independence Measure for children and youth selfcare, *TONI-4* Test of Nonverbal Intelligence, *CP* Cerebral Palsy, *GA1* glutaric aciduria type 1

^a^drop out

### Computer-enhanced upper limb rehabilitation system (YouGrabber)

**Fig. 1:**
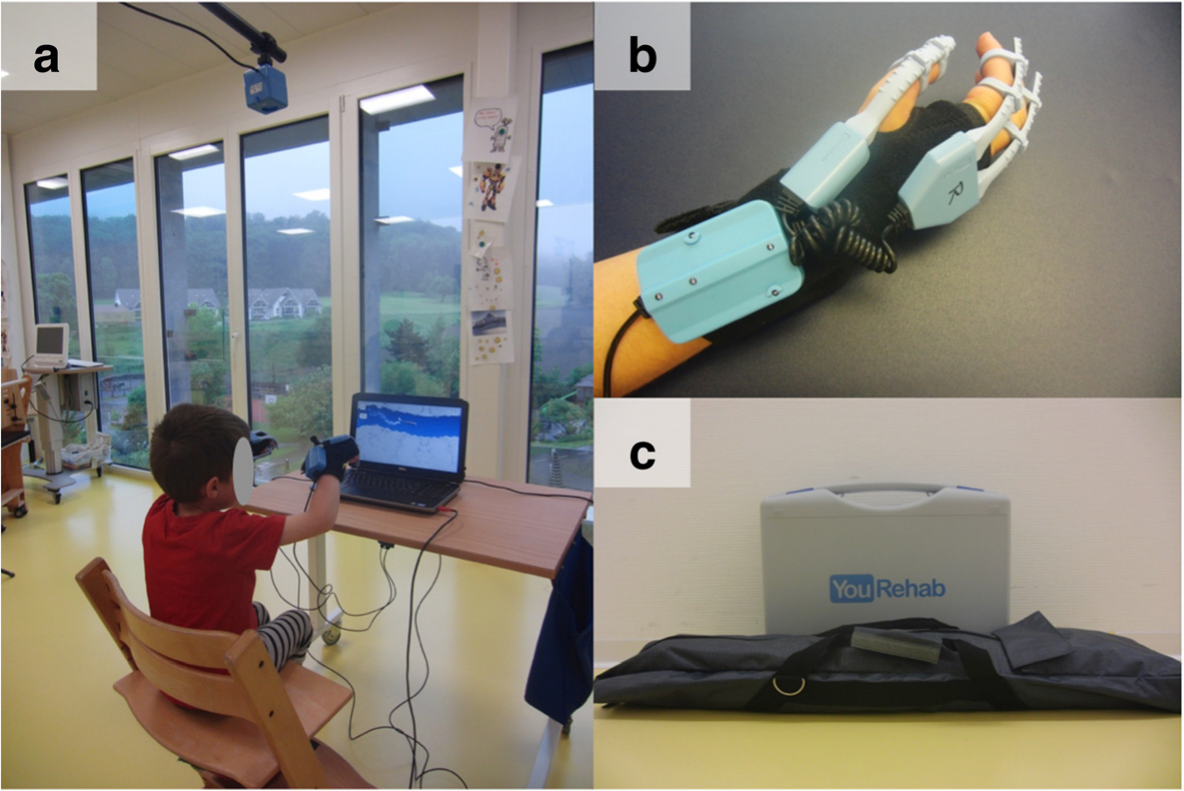
The portable YouGrabber system. **a** A patient playing the Airplane game on the portable YouGrabber system. **b** The complete data glove with sensor-“box”, bending sensors, and vibrating units attached to the size fit neoprene glove. **c** The complete equipment packed for “take away”

The portable YouGrabber system is a computer-enhanced upper limb training system (Fig. 1a). “Boxes” containing sensors were attached with Velcro to the size fit neoprene gloves. A camera mounted above the child tracked the infrared lights from these “boxes” to record the position of the hand in space. The “boxes” also contain magnetometers and accelerometers, which record changes in movements while bending sensors detect changes in flexion and extension of the thumb, index and middle finger. The bending sensors are attached to the fingers via silicone rings. Small vibrating units are positioned on the back of the hand to provide haptic vibration feedback (Fig. 1b). The neoprene gloves are available in four sizes (S, M, L, XL) and the silicone rings in six sizes (XS, S, M, L, XL, XXL), which makes the system applicable to children and adolescents aged between 5 and 18 years old.

The whole equipment, including laptop, gloves, cables, etc. fits into a solid suitcase 44x33x14cm and an 89 cm long bag containing the tripod (Fig. 1c).

Depending on the game scenario, single joint movements but also a combination of movements can be trained (i.e. forearm pro- and supination, elbow or shoulder flexion and extension, selective finger movements, grasping, reaching, wrist flexion and extension). The child interacts directly with a game environment and receives haptic feedback.

### Games

In total, eight different exergames are available for upper limb training (Additional file 1: Figure S1). They allow a playful training of different upper limb movements. One of them (i.e. Hamster Splash) has initially been developed for lower limb training but can also be steered with upper limb movements. As our clinical experience showed that children liked the game, we implemented it in the participant’s training plans for upper limb training.

### Training planner

To make the system easier to use for patients and their families, the software consists of an app called “training planner”. With this training planner, therapists can arrange the games at various difficulty levels, steering options, and with different feedback models. In this manner, tailored therapy plans adjusted to the patient’s impairment, motivation and special needs can be saved.

The training plan starts with a calibration of the maximal joint range of motion of the relevant movements or limb positions, respectively. Pictures on the desktop show always one relevant finger, hand or arm position at the time and patients are asked to hold the position while a caregiver presses the spacebar to save the calibration and continue with the next finger, hand or arm position. When the calibration is finished, the actual training begins. Before every game, there is written information on the desktop on how the following game has to be operated. At the end of the game, the patient receives feedback on the game performance.

At home, once the system is installed, the user just needs to start the computer, log in to the user profile and choose the correct training plan.

### User manual

A user manual comes with every system. We adapted the manual exclusively for the home-use, focusing on the target group of children and adolescents and their caregivers. It consists of (i) a brief general explanation of the system and all the material, (ii) the instruction of how the system can be installed, mounted and used at home, (iii) a summary of all the games and what movements are trained playing them, (iv) annotation where in the software helpful information can be found, (v) a short list of problems that could occur and how they can be solved, (vi) an instruction how to connect the system to the internet for remote control, and (vii) an email address and phone number for technical support.

### Intervention and protocol

The feasibility trial was divided into two phases. In phase 1, we recruited children and adolescents directly after a rehabilitation stay were they already trained with the stationary YouGrabber and thus were familiar with this computer-enhanced system. In phase 2, we recruited patients whose discharge from stationary rehabilitation was at least three months ago. We were interested whether these patients were more motivated, had greater problems in device handling, and would achieve a higher amount of training time, as in comparison to patients in phase 1, they might be less familiar with the YouGrabber system.

Before taking the system home, participants came for a standardized instruction session of 45 min that took place at the rehabilitation center in Affoltern am Albis. A human movement scientist explained every step following the user manual. Parents were asked to set up the system and mount the gloves to their child. The human movement scientist gave further explanations or helped parents whenever necessary. At the end of the instruction session, parents performed a demonstration version of the training plan with their child. In this demo-session, every game was played for 30 s, and the human movement scientist explained the aim of the single games and the whole training session as well as which therapeutic goals were pursued with each specific game.

Children and their parents were instructed to train at least 5 times for 30 min during the first week. In the second week, they were allowed to train as much as they wanted. We chose this approach to see whether such an intensive schedule was theoretically feasible and if participants would continue with a comparable amount of training in the second week without any guideline. Following the instruction session, they took the system home for two consecutive weeks.

After the first week, the families were contacted by telephone. They reported how well the system worked (e.g. if no technical problems occurred, if the training plan was too difficult or too easy etc.) and if anything had to be changed. After that, parents connected the system to the Internet and a research member downloaded training data from the first week and adjusted the training plan if necessary.

### Outcome measures

#### Device usage

The YouGrabber automatically records data whenever a game is running. This data was exported in the form of a training report. Furthermore, parents kept a training diary to document each training session: (i) which caregiver, (ii) time needed to prepare the exercise, (iii) duration entire training session, and (v) general remarks (e.g. technical problems, problems with games, etc.). These data were used to quantify the following parameters; (1) total pure playtime (i.e. only the time where the child was actively playing the game, without calibration, beaks, etc.), (2) time per training session (including donning and doffing and so on), (3) total number of training sessions, and (4) general impression of the training session.

### User satisfaction

User satisfaction was evaluated with questionnaires for every caregiver and child. There were questions about usability, motivation, and the general opinion. Participants answered on a Visual Analogue Scale (VAS). The VAS score was given on a 100 mm long line. The most positive answer was always at the right end of the line (at 100 mm) and the most negative one at the left end of the line (at 0 mm). In line with the approach of Huijgen et al. [13], the scale was subsequently grouped into three categories; VAS scores of 30 or less were categorized as not satisfied; those from 31 to 69 as average scores; and those with scores of 70 or more were categorized as satisfied. Additional questions were provided to get further explanations for VAS scores.

### Patient characterization

The MACS [12] characterizes children’s hand functioning on a 5-level scale. Children with MACS level I can handle objects easily and successfully whereas the ones with MACS level V cannot handle objects and have severely limited ability to perform even simple actions.

To assess independence in ADL, nurses routinely score children in our center with the Functional Independence Measure for children (WeeFIM) [14]. The WeeFIM measures children’s performance in daily life and consists of the three domains self-care, mobility, and cognition. For our study, we used the percentage of total scores of the self-care domain (WeeFIM sc) excluding items on bladder and bowel control, as these items do not assess upper extremity function.

Cognitive capacity of participants was assessed with the 4^th^ edition of the Test of Nonverbal Intelligence (TONI-4) [15]. The TONI-4 aims at language-free intelligence testing. Scores are given as age-corrected percentile rank where a rank between the 25^th^ and 75^th^ percentile is considered an average performance. All assessors were not familiar with the study methodology and aim.

### Usability

We tried to solve technical problems ourselves, and contacted the company if problems persisted. To evaluate the usability, the amount and type of support were estimated from the following data: (1) number of support requests, (2) sort of problems, (3) number of updates and bug fixing by the company, and (4) additional support of the caregivers at home.

### Data analysis

Device usage and amount of support were reported with descriptive statistics. If two caregivers answered for one child, we calculated a weighted mean for the VAS score. Normality of distribution was tested with the Shapiro-Wilk test. Within-subject comparisons (i.e. number of training sessions in week 1 versus week 2) were examined using paired *t*-test for normally distributed data and with the Wilcoxon signed rank-test for non-normally distributed data. Between-group differences (e.g. pure playtime of participants in phase 1 versus phase 2) were analyzed using the unpaired *t*-test for normally distributed, and the Mann–Whitney-*U* test for non-normally distributed data. The relationship of age with device usage, VAS scores, and device usage with clinical characteristics of patients was examined using Pearson (normal distribution of data) or Spearman (non-normal distribution of data) correlation coefficients. To interpret the correlation coefficient we used the following definitions: 0–0.25 (no or little relationship), 0.25–0.50 (fair degree), 0.50–0.75 (moderate to good relationship), 0.75–1.00 (very good to excellent) [16].

All statistical tests were performed using SPSS (Version 22, IBM Corporation, New York, USA). We used pairwise deletion for missing data. For all analyses, alpha was set at 0.05.

## Results

We examined 42 patients for eligibility, which was confirmed for 27 patients. We received informed consent from 16 families, of which one family withdraw because of social issues before study onset. Fifteen participants were included in the study. One of the participants (ID 13) never trained with the device and, because of this poor compliance, was not included in the analyses.

In twelve cases, parents and their children were instructed at the rehabilitation center. For two families, it was difficult to come to the rehabilitation center for the instruction session. Therefore, a human movement scientist visited them at home and installed the device together with the patients and caregivers in the home setting.

### Device usage

One session (including glove mounting, calibration, breaks, etc.) lasted on average 41.5 ± 8.3 min. The mean total pure playtime in the 2 weeks was 186.7 ± 48.0 min. Children trained a median of 4 sessions (range 2–6) in the first, and 2.5 sessions (range 0–6) in the second week. In total, they trained 7 sessions (median; range 4–12). All participants together performed 99 training sessions throughout the study.

**Fig. 2:**
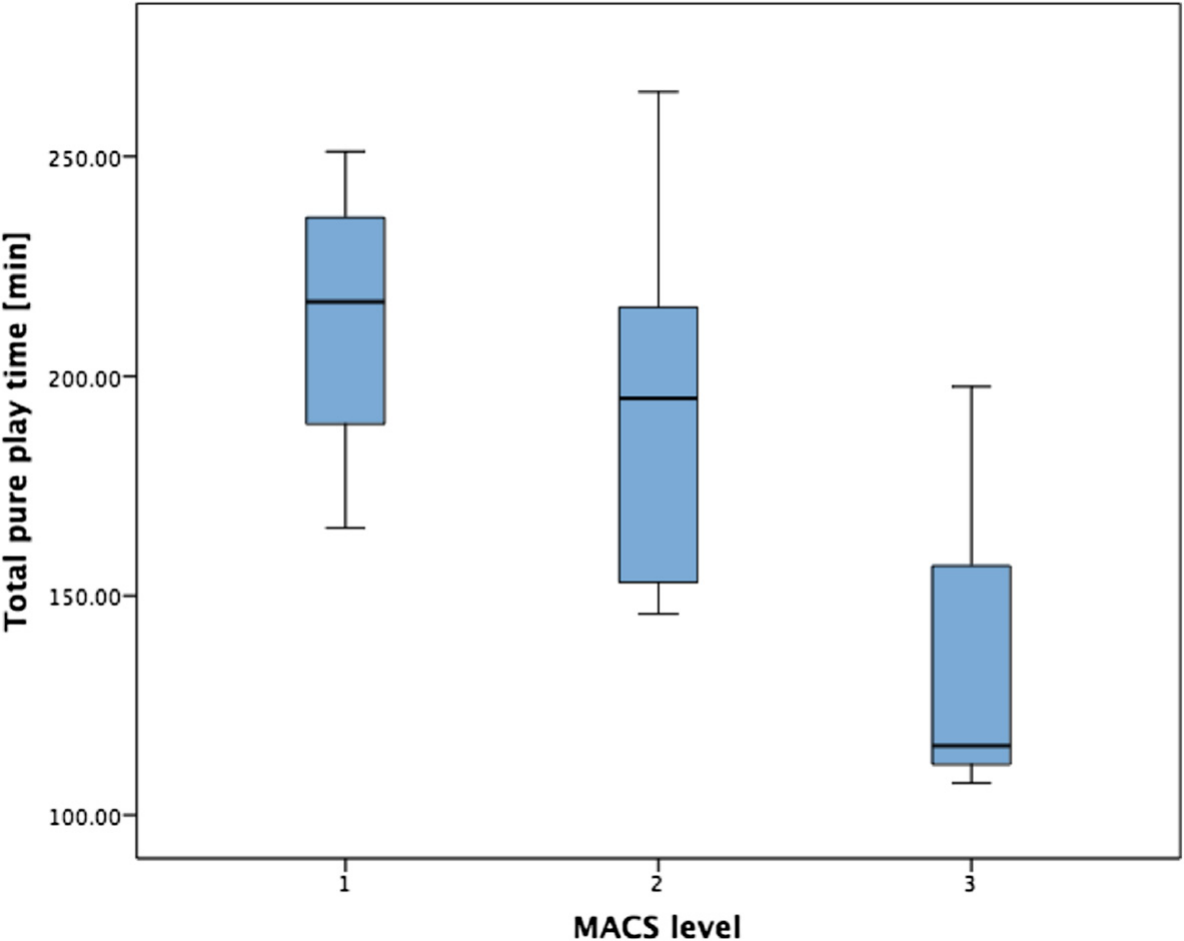
Relationship between manual ability and total pure playtime. The boxplots of total pure playtime for the different Manual Ability Classification System (MACS) levels show that more severely affected children and adolescents tend to train less with the system

Despite that we found a significant negative correlation between manual ability (i.e. MACS levels) and total pure playtime during the 2 weeks (ρ =–0.54, *p* < 0.05), pure playtime did not differ significantly between children with different MACS levels (post-hoc Kruskal-Wallis test, Fig. 2).

Participants with higher WeeFIM self-care scores achieved a higher number of trainings in the second week (*ρ* = 0.82, *p* < 0.001) and total number of training sessions (*ρ* = 0.67, *p* < 0.05). There was no significant correlation between nonverbal intelligence (i.e. TONI-4 scores) and device usage.

We could read from the training diaries that in 68 of the 99 sessions (i.e. 67 %), participants and their caregivers encountered problems they attributed to malfunctioning of the system. Problems with the calibration occurred in 16 % of all sessions while issues with the movement tracking occurred in 32 % of all sessions. Additionally, in 36 % of all sessions, caregivers reported that at least one game “did not work properly” or “caused problems” without further specification. In 11 sessions, the computer had to be restarted.

### User satisfaction

#### Participant questionnaire

VAS scores given by participants are depicted in Table 2.

**Table 2:** User satisfaction (participants)

Questions							# Answers in VAS category		
0 = negativ end, 100 = positive end		N	Min	Max	Mean	SD	≤30	31-69	≥ 70
1	How interesting was the training with the YouGrabber?	13	30	100	70.9	21.7	1	5	7
2	Did you feel discomfort or pain during the training?	13	32	100	76.1	27.7	0	5	8
3	Was the training length appropriate?	13	4	100	57.9	34.4	2	6	5
4	Would you like to continue with such training?	13	2	99	53.3	35.7	3	4	6
5	What is your general impression of the System?	13	25	92	66.1	22.7	1	5	7

Abbreviations: *Min* minimum, *Max* maximum, *SD* standard deviation, *VAS* score categories, *≤ 30* unsatisfied, *31-69* average, *≥ 70* satisfied

Only one patient adjudged the YouGrabber training as not satisfactorily interesting (VAS of question 1 ≤ 30). Some patients reported discomfort or pain caused by the silicone rings and the neoprene gloves, which appeared too tight around the metatarsophalangeal joints. Almost half of the patients rated the training time as too long. Three children would not like to continue with the YouGrabber training (VAS of question 4 ≤ 30).

#### Caregiver questionnaire

VAS scores given by parents are shown in Table 3.

**Table 3:** User satisfaction (parents)

Questions							# Answers in VAS category		
0 = negativ end, 100 = positive end		N	Min	Max	Mean	SD	≤ 30	31-69	≥ 70
1	How complicated/time consuming was the installation of the system?	12	47	100	74.2	19.5	0	4	8
2	How easy was the use of the YouGrabber system?	14	48	98	80.9	15.0	0	4	10
3	How often did technical problems occur?	14	17	100	57.4	27.0	4	3	7
4	How useful was the user manual?	12	59	98	85.4	13.5	0	2	10
5	How useful were the instructions given to you before the two week trial?	14	68	100	92.3	9.6	0	1	13
6	Did your child need to be encouraged to start with the training?	14	25	98	66.5	26.5	3	3	8
7	Did your child need to be encouraged to complete the whole training session?	14	6	98	58.6	35.7	5	2	7
8	How big do you estimate the therapeutic benefit or the system?	14	26	100	71.8	22.0	1	5	8
9	What is your general impression of the system?	14	50	98	85.6	12.4	0	1	13

Abbreviations: *Min* minimum, *Max* maximum, *SD* standard deviation, *VAS* score categories, *≤ 30* unsatisfied, *31-69*, average, *≥ 70* satisfied

Four parents notified the too frequent occurrence of technical problems (VAS of question 3 ≤ 30). Three and five parents perceived the amount of motivation their children needed to start with the training or to continue with the same, respectively, too high (VAS of questions 6 and 7 ≤ 30).

### Relationship of age with device usage and VAS scores

**Fig. 3:**
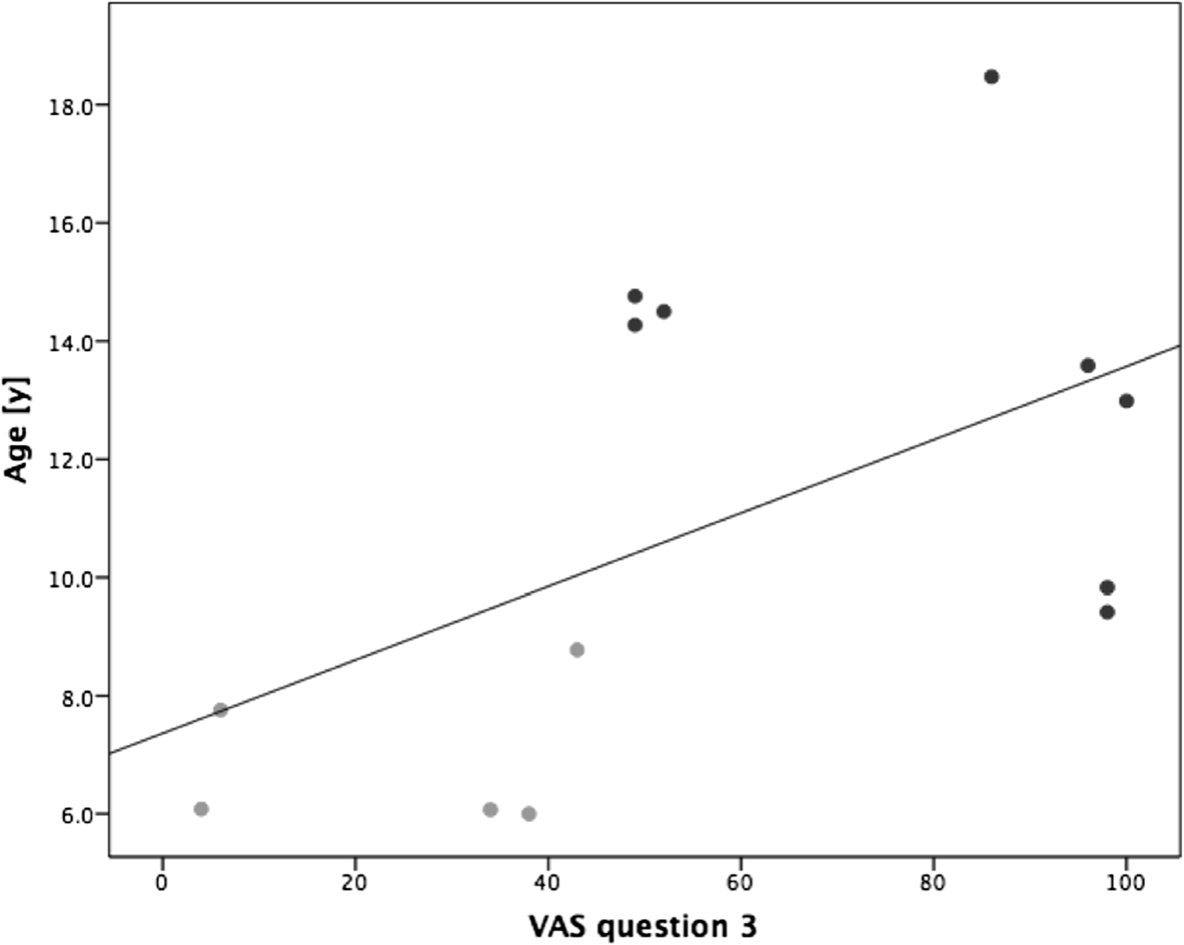
Correlation of VAS score of patient questionnaire, question 3, with age. Age correlated with question 3 “was the training length appropriate?” of the patient questionnaire. VAS scores could vary between 0 – not at all, and 100 – absolutely. Clear dots: patients under 9 years, dark dots: patients over 9 years

There was no correlation between age of participants and the amount of effective playtime or number of training sessions. We found, however, a strong negative relationship between the given VAS score for the therapeutical benefit (rated by parents) and the children’s age (*ρ* =–0.73, *p* < 0.05). Moreover, age correlated moderately with the VAS scores of the question whether children perceived the training time as accurate (*ρ* = 0.55, *p* = 0.05, Fig. 3).

### Differences between study phases

No differences in device usage (e.g. pure playtime) were found comparing the outcomes of study phase 1 with study phase 2. Only the VAS score of the question “how easy was the use of the YouGrabber system?” (question 2 rated by parents) was significantly lower in the group of study phase 2. There were no differences in age, MACS level, WeeFIM, and TONI-4 scores between patients included in the different study phases.

### Usability

Throughout the study, we needed support from the company 24 times. The main problems were; (i) the training plan did not start, (ii) single games did not start (screen became white), (iii) changes in the training planner could not be saved, or (iv) updates running in the background turned computers very slow. During the study, the company undertook all systems two general updates. Four times, one or several computers had to be brought to the company for specific bug fixing.

Additionally to the scheduled phone call at the end of the first week, 10 out of the 15 participating families contacted us at least once for supplementary telephone support.

## Discussion

The aim was to examine the feasibility and usability of home-based computer-enhanced upper limb training for children and adolescents with central motor disorders. Previous studies with the YouGrabber system have been conducted in rehabilitation centers and with a therapist assisting the therapies [10, 17]. To our knowledge, this is the first study where the YouGrabber system for upper limb rehabilitation is used in the home setting.

The main results showed that children achieved a median of 7 training sessions over the 2 weeks with a high total pure playtime. Even though in more than half of the sessions technical problems occurred, children and parents were, in general, satisfied with the system. Children with higher functional levels trained more often and achieved a higher pure playtime than more severely affected patients. Throughout the study phase, support by the company and our project members was required.

### Device usage

Device usage might reflect the acceptability of the system and is in the light of motor learning, and therapy intensity is of uttermost importance in achieving high enough levels of active participation.

Participants achieved a mean training time of 287.1 min over the whole period. With a mean of 186.7 min, the total pure playtime was quite high in our study. Weightman et al. [18] conducted a feasibility study with a computer-assisted system for home-use where 18 children with CP trained a mean of 75 min over a period of 4 weeks (i.e. 37.5 min in 2 weeks). In a study of Rios et al. [19] four children with CP performed home-based NeuroGame Therapy over a period of 5–6 weeks. They achieved a mean training time of 1.76 h per week (i.e. 213 min in 2 weeks). However, in these two studies it does not become clear whether only pure active playtime or also rest periods were included in the duration they reported. In a home-based feasibility trial of Huber et al. [20], three adolescents with CP trained a mean of 28.5 h over a period of 6 to 10 months with gaming technology addressing hand impairments (i.e. 85.5 min in 2 weeks). However, participants from the study of Huber et al. [20] trained over a period that was 12 to 20 times longer than our feasibility trial.

To make different studies comparable, but also as an indication of the efficiency of a training system, it is important to distinguish between training duration and pure playtime. In our study, we suspect the large difference between these two measures arising from two different sources. On the one hand, donning and doffing, calibration, and breaks between individual games are reasonable causes for additional training time. On the other hand, technical problems, lack of motivation, and difficulties in mounting gloves can cause prolonged training times leading to a low proportion of pure playtime to total training time indicating inefficiency.

Children did not achieve the goal of 5 training session in the first half of the trial. In the second week, they trained even less frequently. The most common explanations were the hot summer weather (children preferred staying outside) and the rather stressful period before the summer holidays. These findings indicated that therapists should carefully select the periods when home training should occur, taking into account various personal and environmental factors such as reported here.

The higher their manual ability (i.e. lower MACS level), the more pure playtime children achieved (Fig. 2). Likewise, the more independent children were in their ADL (i.e. WeeFIM self-care score), the more they trained. Similarly as with younger age, the training might be rather demanding for children with higher MACS levels and lower WeeFIM self-care scores. Furthermore, the training may be easier to organize for more independent children, as they need less help and donning and doffing might be easier and quicker.

In 67 % of all sessions, parents reported the occurrence of problems they perceived as not self-induced. The main problems were the movement tracking not working properly and the calibration causing difficulties. These two problems are highly related, as the movement tracking cannot work well if the calibration is not performed correctly. We recommend therefore improving and simplifying the calibration procedure.  In one-ninth of all sessions, restarts were inevitable because of suddenly interrupting updates, screen turning white or freezing, or because the gloves were suddenly not detected anymore by the system. In two sessions, caregivers reported that one or several games blocked, without further information if the system required a restart. Such software-bugs should be solved before distributing the system to outpatient users.

### User satisfaction

As all mean VAS scores given by participants and their caregivers fell in the “average” (6/14 questions) or “satisfied” (8/14 questions) categories, we can conclude that regarding user satisfaction, participants and their parents rated the YouGrabber system for home-use as highly acceptable. However, several individual scores were very low and fell in the category “not satisfied”. We suggest that mean scores in the category “satisfied” can be accepted without further adaptations of the issue, whereas for those in the category “average”, efforts should be made to find reasons for the lower scores and to improve the system regarding this issue. For questions with mean scores in the category “not satisfied”, it is crucial to address the underlying problem before further applying the system to patients.

#### Patient questionnaire

Reasons for patients evaluating the YouGrabber training rather boring were: (i) few games (especially lack of games targeting training of fine motor skills and ADL), (i) little variation, (iii) unsatisfactory art design, and lack of challenge and competition.

Although the silicone rings were sometimes uncomfortable, and the neoprene gloves were too tight at the distal end, no participant rated the discomfort/pain equal or below 30. One patient had back pain because of the prolonged sitting. However, none of these events was categorized as a serious adverse event.

Two girls, both below 9 years (ID 10 and ID 15), said the training time was way too long (VAS of 6 and 4, respectively) and they would not like to continue with the YouGrabber training (VAS of 6 and 2, respectively). For the older of these two girls, it was too much to train besides the school and in her free time she preferred to do other things than training the upper extremities. The younger girl said that sometimes the system did not work and therefore, she would not like to continue with the training. She also mentioned, however, that in the future she could imagine training for a longer period. Indeed, the mothers of both girls had to encourage their daughters very much to complete the training sessions (VAS of 14 and 6, respectively). Another patient (ID 3) who did not like to continue with the training (VAS score of 5), explained that the system never worked properly and that therefore he was happy to return it. Also, his mother reported frequently occurring technical problems (VAS score of 25).

A nine-year-old boy (ID 7) rated the training with the YouGrabber system as not interesting enough. He would have liked more games with more variation. He also had a rather unsatisfied impression of the whole system (VAS score of 25). Interestingly, he would still have liked to continue with the training for one or two more months showing the discrepancy between the acceptance of the system itself and the general motivation for home-based training.

#### Caregiver questionnaire

The worse mean VAS score of the caregiver questionnaire was the one about the frequency of technical problems. This is in line with outcomes of the feasibility study by Huber et al. [20] where the average score for the question about technical problems was 2.8 of 7 (1-frequent problems, 7-no problems). In our study, four VAS scores of the corresponding question were below 30. On the other hand, 7 scores were equal or above 70. This is somewhat surprising when considering that in the training diaries parents reported technical problems in 67 % of the sessions. However, justifications for the VAS scores were in line with the notifications we found in the training diaries.

The other questions with a mean VAS score of less than 70 were the two about motivation. Children needed to be less motivated to begin with a training session than for its continuation. Reasons, why children were not very motivated to begin with the training, were the nice weather (ID 4), social issues (ID 4, ID 8), and technical problems in the previous session (ID 3, ID 4). Reasons for the need of extrinsic motivation throughout one training session were the lack of challenge and bonus at the end of the games (ID 2), technical problems within the session (ID 3, ID 10, ID 15), and the length of the training (ID 9, ID 10, ID 15). The mother of a nine-year-old boy (ID 12) explicitly reported technical problems having a strong influence on how much her son needed to be motivated. Nevertheless, she gave VAS scores of 77 and 46 for the questions about motivation.

Only the mother of the girl with neuropathy (ID 2) gave a VAS score below 30 for the question about the therapeutical benefit. She said the system was not challenging enough for her daughter.

From the VAS scores of the two questionnaires, it becomes clear how large the influence of technical difficulties especially on the motivation of the children is. Error-prone systems result quickly in frustration and reduced motivation and therewith decrease compliance. Another issue that affects children’s motivation are the games. Children reported a lack of challenge and bonus at the end of the game and criticized the quality of the graphics. To address this issue, also known from the stationary setting, YouRehab newly guarantees to launch two new games every year (personal communication with the CEO of YouRehab).

Overall, however, the YouGrabber system was rated as easy to use, and participants and their caregivers rated the system as good. Similarly did participants in the CP telerehabilitation study of Huber et al. [20].

### Relationship of age with device usage and VAS scores

Parents of younger children rated the therapeutic benefit of the system higher than those of older children. One explanation could be that to keep them motivated, the younger patients need a playful training while for the older ones a more specific ADL-training is possible and might be more goal-oriented. Furthermore, for older children, the games were possibly not variable and challenging enough while for the younger ones it was rather demanding. The finding that age correlates with the acceptability of the training length supports this theory. All children younger than 9 years perceived the time of one session as too long (Fig. 3). Nevertheless, there was no correlation between effective pure playtime or number of training sessions with age. Perhaps this also indicates that for younger patients too much time was needed for donning and doffing. Furthermore, parents might have motivated their children to hang on until the end of the session.

### Differences between study phases

There were no differences in age or clinical characteristics between patients participating in phase 1 versus phase 2. So, despite the small sample size, we concluded that participants of the two study phases were more or less comparable.

The only significant difference in outcomes between study phases was that parents of children in study phase 2 rated the system as being more complicated to use compared to those of phase 1. Families of phase 1 took the YouGrabber system at home, right after a rehabilitation stay where children already trained with the stationary YouGrabber system. Therefore, these children might have been more familiar with the system than children from study phase 2. Probably, they were also better in helping their parents regarding glove mounting and handling of the system. Additionally, remembering therapist’s feedback from the recent rehabilitation stay, they might have had clearer ideas of successful movement execution than their peers from study phase 2.

### Usability

One of the advantages of home-based therapy are the reduced treatment costs while the amount of training remains the same or even increases. Due to different technical problems occurring throughout the study and subsequently, the time spent to solve the problems and support participating families, much more resources were used as we originally expected. Consequently, the YouGrabber system, as it is right now, does not meet the requirements of being cost efficient.

In other feasibility studies with a comparable patient group, it is not stated how much support they provided throughout the study phase. Weightman et al. [18] state that little support was needed, once the system was installed at home. They further mention home visits were mainly to adjust task difficulties. However, there is no quantification of the amount of support.

### Limitations and outlook

The length of the training phase was rather short (i.e. 2 weeks). However, most children would have liked continuing with the training and they, as well as their parents, had a good general impression of the whole system. We conclude that families would accept the system also for a longer time-span.

Additionally, in our feasibility trial we performed no clinical pre and post measures and, therefore, cannot state whether such home-based YouGrabber training induced upper limb improvements. Previous studies with the stationary system, however, suggest that training with the YouGrabber (or its early version, the “PITS”) is intense and evokes functional upper limb improvements [10, 17].

Huber et al. [20] stated that remote computer monitoring showed when participants did not train anymore and helped to motivate them to resume with the therapy. We conducted only one telephone call after one week to eventually adjust the training plan. We provided no additional motivation.

According to our inclusion criteria, children and adolescents with congenital or acquired brain lesions and MACS levels from I to IV could participate. Finally included in our study, however, were almost exclusively patients with CP and MACS levels I to III. This limits the generalizability of our findings to patients with other diagnoses and MACS level IV. Other factors like age and cognitive functioning reflect well the heterogeneous patient populations seen on a daily basis in rehabilitation.

Summing up, for future studies, we suggest planning a longer intervention phase and performing pre and post assessments. A closer attendance of families at the beginning might help for early detection of problems and throughout the study to motivate patients in case of discontinuous training.

Patient questionnaires showed that the training length of one session (mean 41.5 min) was too long for younger participants (Fig. 3). On the other hand, for rather independent patients with higher functional levels, it might be easier to perform more training sessions and achieve higher pure playtime. Therefore, we recommend taking into account children’s age and manual ability as well as independence in daily life in the planning of future interventions.

Before it comes to clinical implementation of such training tools for home-use (i.e. other than in the research setting), important questions have to be addressed. Although this study addresses some aspects of the clinical utility of the YouGrabber system in the home setting (i.e. its acceptability by children and their parents and usability), for a comprehensive evaluation of a new technology, other aspects of clinical utility should not be neglected [21]. Firstly, its effectiveness, efficacy, and relevance should be investigated in clinical trials. Secondly, the acceptability by clinicians and the society should be tested. And thirdly, financial aspects need to be clarified (e.g. who would cover the costs of purchasing or renting such systems, and who would reimburse the costs of therapeutical instructions and supervision or technical support?).

## Conclusion

The results on the amount of device usage are very promising for future applications of the YouGrabber system at home as a motivating training tool to complement usual care and therewith augment training frequency and intensity in children and adolescents with neuromotor disorders. We can conclude that the YouGrabber system is easy to use, well accepted, and beneficial (as rated by the parents) for home-based upper limb training. Nevertheless, the frequency of technical problems was very high in this trial. A technically more robust system, combined with additional attractive games, likely results in higher patient motivation and better compliance, which would reduce the need for parents to motivate their children extrinsically and allow for clinical trials to investigate the effectiveness of the system.

## Additional file

Additional file 1: Figure S1: Games of the portable YouGrabber system. Eight games are available for the YouGrabber system for home-use. For many games, different control options are available. In the figure we depicted the most common ones. (PNG 901 kb)

## Abbreviations

ADL: Activities of daily living
CP: Cerebral Palsy
MACS: Manual Ability Classification System
TBI: Traumatic brain injury
TONI-4: Test of Nonverbal Intelligence 4^th^ edition
VAS: Visual Analogue Scale
WeeFIM: Functional Independence Measure for children
