# Supplementary figures and images for: Preparing a neuropediatric upper limb exergame rehabilitation system for home-use: a feasibility study
Source: J Neuroeng Rehabil. 2016 Mar 23;13:33. doi: 10.1186/s12984-016-0141-x (PMC4806437; doi:10.1186/s12984-016-0141-x)

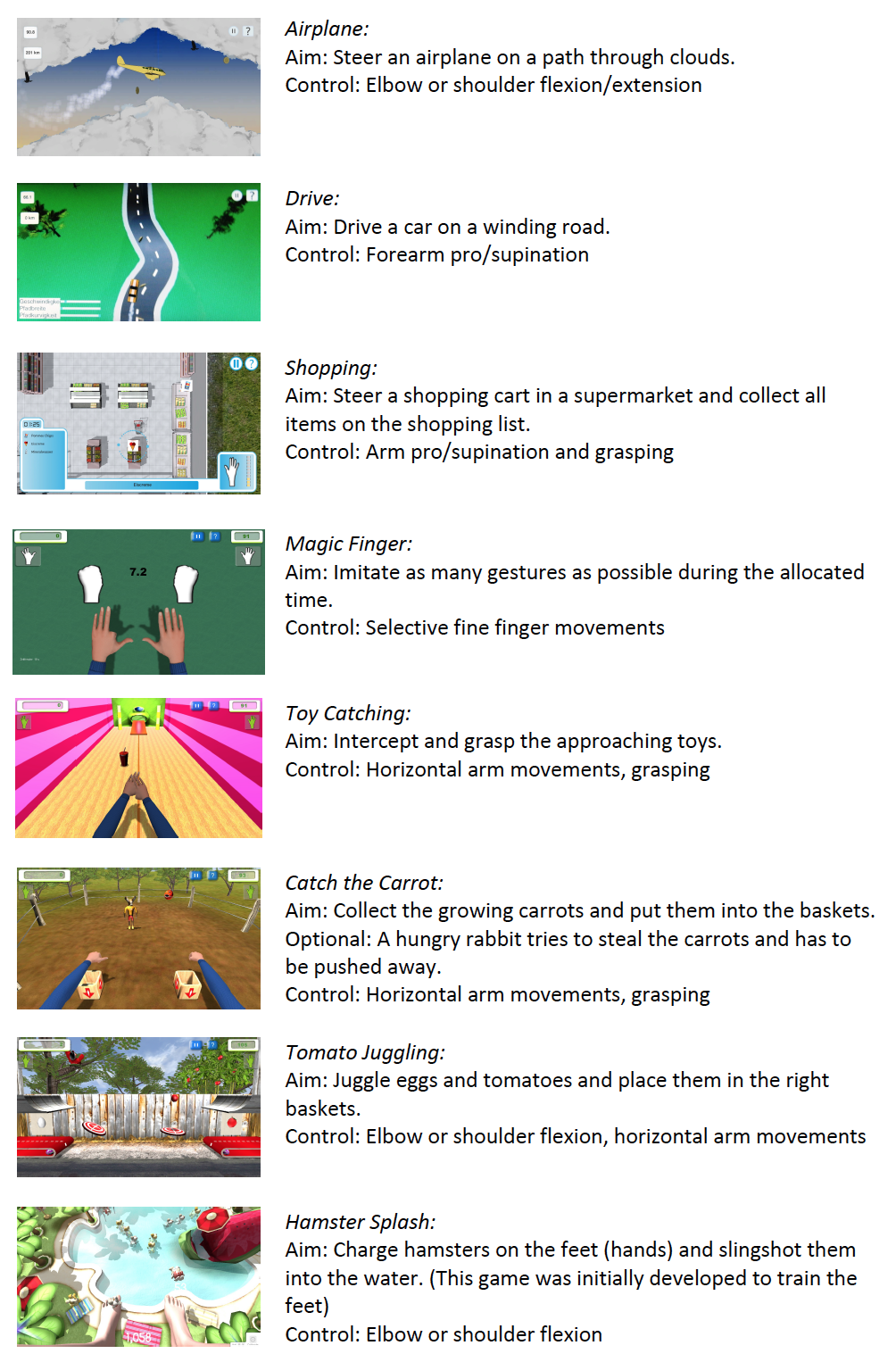

Supplement: Additional file 1: Figure S1: — Games of the portable YouGrabber system. Eight games are available for the YouGrabber system for home-use. For many games, different control options are available. In the figure we depicted the most common ones. (PNG 901 kb) [file 12984_2016_141_MOESM1_ESM.png]
